# The gender gap in relation to happiness and preferences in married couples after childbirth: evidence from a field experiment in rural Ghana

**DOI:** 10.1186/s41043-017-0084-2

**Published:** 2017-03-15

**Authors:** Yusuke Kamiya, Bright Akpalu, Emmanuel Mahama, Emmanuel Kwesi Ayipah, Seth Owusu-Agyei, Abraham Hodgson, Akira Shibanuma, Kimiyo Kikuchi, Masamine Jimba, Ghana EMBRACE Implementation Research Project Team, Yoshiharu Yoneyama, Ebenezer Appiah-Denkyira, Masamine Jimba, Abraham Hodgson, Gloria Quansah Asare, Evelyn Korkor Ansah, Junko Yasuoka, Keiko Nanishi, Akira Shibanuma, Kimiyo Kikuchi, Sumiyo Okawa, Margaret Gyapong, Sheila Addei, Vida Kukula, Doris Sarpong, Clement Narh, Seth Owusu-Agyei, Kwaku Poku-Asante, Charlotte Tawiah, Yeetey Enuameh, Kwame Adjei, Emmanuel Mahama, Abraham Rexford Oduro, John Williams, Cornelius Debpuur, Francis Yeji, Evelyn Sakeah, Peter Wontuo, Akiko Hagiwara, Sakiko Shiratori, Yusuke Kamiya

**Affiliations:** 1 Faculty of Economics, Ryukoku University, 67 Tsukamoto-cho, Fukakusa, Fushimi-ku, Kyoto City, 612-8577 Japan; 2 Kintampo Health Research Centre, P.O. Box 200, Kintampo, Brong-Ahafo Ghana; 3 Research and Development Division, Ghana Health Service, MB 190 Accra, Ghana; 4 Department of Community and Global Health, Graduate School of Medicine, The University of Tokyo, 7-3-1 Hongo, Bunkyo-ku, Tokyo, 113-0033 Japan; 5 Institute of Decision Science for a Sustainable Society (IDS3), Kyushu University, 3-1-1, Maidashi, Higashi-ku, Fukuoka City, 8128582 Japan

**Keywords:** Gender, Marriage, Happiness, Preferences, Childbirth, Economic experiment, Ghana

## Abstract

**Background:**

How does the gap in preferences between married couples affect their happiness after childbirth? Are couples that share similar preferences happier? In recent years, gender, marriage, and happiness have been considered to be key issues in public health research. Although much research has examined the happiness status of married couples, practically no study has explored the gender gap in relation to happiness and the preferences of married couples after childbirth. Therefore, our study was conducted to assess the association between the preference gap and the happiness status among married couples in the afterbirth period.

**Methods:**

We conducted a field experiment in rural communities in the Brong-Ahafo region of Ghana. Participants were 80 married couples who had experienced childbirth within 2 years prior to the survey. As preference indicators, we measured trust, reciprocity, altruism, and risk lovingness through an economic experiment. Then, we assessed how, for a couple, the gap between these preferences affected their happiness.

**Results:**

Wives’ happiness was positively associated with the absolute value of the gap in risk lovingness between a couple (OR = 4.83, *p* = 0.08), while husbands’ happiness was negatively associated with the gap in trust (OR = −3.58, *p* = 0.04) or altruism (OR = −3.33, *p* = 0.02). Within a couple, wives felt greater happiness than their husbands if there was a wider gap in trust (OR = 6.22, *p* = 0.01), reciprocity (OR = 2.80, *p* = 0.01), or risk lovingness (OR = 3.81, *p* = 0.07).

**Conclusions:**

The gender gaps in the preference indicators were found to be closely associated with the happiness levels between married couples after childbirth. For the further improvement of maternal and child health, we must consider the gender gaps between couples in relation to happiness and preferences.

## Background

In recent years, gender, marriage, and happiness have been considered to be key issues in public health research [1–5]. In particular, it is important to examine the happiness status of married couples, often measured by subjective well-being or life satisfaction, in the afterbirth period. This is because higher subjective well-being or lower life stress is associated with a lower risk of postpartum depression among couples after childbirth [6–9]. As postpartum depression has potentially negative consequences on child growth and development [7, 10], the happiness status of married couples after childbirth must be closely investigated.

A growing body of empirical research has examined the happiness status of married couples [11–13]. Nevertheless, practically no study has explored happiness in relation to couples’ preferences. Preferences, which include trust, altruism, cooperation, inequality aversion, and risk preferences (risk lovingness or risk aversion), have been examined as potential determinants of health-related behaviors or outcomes [14–16]. For example, risk aversion has been negatively associated with cigarette smoking, heavy drinking, and obesity in the USA [14] while in Spain, altruism, fairness, trust, and reciprocity have not been associated with high body mass index (BMI) [15].

However, the association between the gender differences is still not clear in regard to preferences and the happiness status between couples. Therefore, our study was conducted to assess the association between the preference gap and the happiness status for married couples during the afterbirth period. Among the preferences, our study focused primarily on trust, reciprocity, altruism, and risk lovingness.

## Methods

### Study design and target population

Under a cross-sectional design, we conducted an economic experiment and questionnaire survey in eight rural communities in the Kintampo North Municipality of the Brong-Ahafo region, Ghana from December 2012 to January 2013. We performed the study as part of the Ghana Ensure Mothers and Babies Regular Access to Care (EMBRACE) Implementation Research Project, which stemmed from a collaboration between Ghana and Japan [17]. We collected the data using the Kintampo Health Demographic Surveillance System (KHDSS), which mainly covered populations in the Kintampo North and Kintampo South districts of the Brong-Ahafo region. Using the KHDSS database, we targeted married couples from eight rural communities in Kintampo North Municipality; for each of these couples, the women were aged between 18 and 49 years and had experienced a birth within the 2 years prior to the study. A search of the database returned 199 women who met these criteria; we then selected 80 women as our sample population using a simple random sampling method. Considering that the mean value of life satisfaction (1: completely dissatisfied to 10: completely satisfied) is 6.09 for adult females in Ghana (a value obtained by Wave 5 of the World Values Survey (2007) [18]), while also allowing for a tolerable error of 5% (0.3045), a population size of 199, and a 5% confidence interval, the minimum sample size was calculated to be 64. Thus, our sample size was a suitable representative of eligible women in the target communities.

### Economic experiment

In the experiment, participants played a standard version of a one-shot trust game, a dictator game, and a risk game. Field instructors gave participants detailed instructions for each game in the local language. In each game, a participant received 10 pieces of “onga,” which is a seasoning used for cooking, as tokens for the game. We used *onga* instead of cash because real money would violate ethical practices. Moreover, participants understood its monetary value.

In the trust game, we measured the level of trust and reciprocity the participants possessed. We applied a method designed by Berg, Dickhaut, and McCabe [19], in which every participant played the game in turn as both a sender and a receiver. As an initial endowment, the sender was given 10 pieces of *onga*. The amount that the sender chose to send to the receiver was tripled and transferred to the receiver. Then, the receiver decided how much they wanted to return to the sender. Here, we defined “trust” as the proportion sent by the sender (out of their endowment) and “reciprocity” (sometimes referred to as “trustworthiness”) as the proportion returned by the receiver out of the amount received.

In the dictator game, we measured levels of altruism. The participant, as a dictator, was given 10 pieces of *onga*, and then, the dictator decided how much of this endowment to split with another participant, a receiver. We defined “altruism” as the proportion donated to the receiver by the dictator. Unlike the trust game, the receiver simply receives the amount decided upon by the dictator and does not return any *onga*. This means that the dictator does not consider possible reciprocity from the receiver when deciding on the amount to give. Consequently, the dictator game can only assess the altruism of the participants who plays the role of the dictator.

In the risk game, we measured the level of risk lovingness. Similar to the previous games, a participant was initially given 10 pieces of *onga* as an initial endowment, and they decided how much of this endowment to bet, similar to a lottery. They rolled a six-sided dice. Each number on the dice (1, 2, 3, 4, 5, or 6) corresponded to particular odds (0, 0.5, 1, 1.5, 2, or 2.5, respectively). After rolling a dice, the amount bet by the participant was multiplied by the corresponding odds. Then, the calculated amount became the pay-off for each participant. The participants understood the corresponding odds for each number on the dice before they bet. We defined “risk lovingness” as the proportion bet by the participant.

On the same day as the economic experiment, we conducted a questionnaire survey for all of the participant couples individually. The questions included the topic of the basic socioeconomic characteristics of individuals, couples, and households.

### Statistical analysis

We conducted descriptive and regression analyses. In the descriptive analysis, we reported differences between wives and husbands in regard to preference indicators, happiness, and individual’s, couple’s, or husband’s characteristics that satisfied a statistical significance obtained through paired *t* tests (Wilcoxon signed-rank tests were used for subjective well-being).

To measure each individual’s happiness, we used their subjective well-being as a proxy variable. Specifically, we measured the level of life satisfaction using a Likert-scale ranging from “1” (completely dissatisfied) to “10” (completely satisfied). This self-rating was obtained by presenting each participant with the following the question: “All things considered, on a scale of 1 to 10, how satisfied are you currently with your life as a whole?” [20].

In the regression analysis, we assessed the effects that gaps in preferences between a couple have on their happiness. Accordingly, we performed multivariable regression analyses using the following “gap” measures: first, for each couple (“c,” where *c* = 1,.., 80), we calculated the within-couple gap in relation to four types of preferences (trust, reciprocity, altruism, and risk lovingness).

1 $$ \mathsf{Preference}\ \mathsf{g}\mathsf{a}{\mathsf{p}}_c = \mathsf{Wife}\hbox{'}{\mathsf{s}}_c\ \mathsf{preference} - \mathsf{Husband}\hbox{'}{\mathsf{s}}_c\ \mathsf{preference} $$

Then, we performed ordered logistic regression analyses in order to assess the effects this preference gap has on the happiness levels of wives and husbands.

Second, we calculated the within-couple happiness gap for each couple.

2 $$ \mathsf{Happiness}\ \mathsf{g}\mathsf{a}{\mathsf{p}}_c = \mathsf{Wife}\hbox{'}{\mathsf{s}}_c\ \mathsf{happiness} - \mathsf{Husband}\hbox{'}{\mathsf{s}}_c\ \mathsf{happiness} $$

where Happiness gap_*c*_ >0 shows that a wife is happier than her husband. We also defined the following latent variable:

3 $$ \mathsf{Happiness}\ \mathsf{g}\mathsf{a}{{\mathsf{p}}_c}^{*}=\mathsf{1}\kern0.5em \mathsf{if}\kern0.37em \mathsf{Happiness}\ \mathsf{g}\mathsf{a}{\mathsf{p}}_c > \mathsf{0},\ \mathsf{otherwise} = \mathsf{0} $$

In the analysis, we performed logistic regression analyses and ordered-logistic regression analyses using (3) and (2) as outcome variables, respectively. For these regressions, we controlled for village-level clustering effects by calculating cluster-robust standard errors. As control variables, we used the participants’ ages, educational attainments (none, primary, middle, higher), and religion (Catholic, Muslim, Charisma, Traditional African, other) at the individual level and the number of living children and household asset quartile (highest, upper middle, lower middle, lowest) at the couple/household level. Regarding household assets, we created a composite asset index using a principal component analysis based on possession of physical assets, such as radios, televisions, refrigerators, fans, bicycles, and livestock. We selected these control variables by referring to past research in Ghana [21–23], which clarified that socioeconomic status, religion, and household characteristics are important predictors of an individual’s happiness.

In both descriptive and regression analyses, we used 10% as the statistical significance level, not the conventional 5% level. This was because of the relatively small sample size in this study. In the tables, we presented the significance level either at the 1, 5, or 10% level so that readers can understand the strength of the statistical significance.

## Results

### Sample characteristics

Table 1 shows the sample statistics of the 80 participating couples. A statistically significant gap existed in mean ages between couples: wives (29.4 years) and husbands (38.1 years). For husbands, educational level was slightly but non-significantly higher than that of wives: 45.0% of wives and 37.5% of husbands did not finish primary education, whereas 5.0% of wives and 10.0% of husbands possessed a higher education. There were more Catholic (33.8 vs. 28.8%), Muslim (22.5 vs. 21.3%), and Charisma (8.8 vs. 7.3%) believers among wives than husbands, respectively. Traditional African religion was more common among husbands (18.8%) than wives (7.5%) (*p* < 0.01). The mean years of marriage was 8.8 years, and the mean number of children was 3.9.

**Table 1:** Sample statistics

	Wife				Husband				Difference
	Mean	S.D.	Min	Max	Mean	S.D.	Min	Max	
Happiness									
Subjective well-being (SWB)	5.6	2.1	1	10	5.8	2.3	1	10	−0.2
Wife’s SWB > husband’s SWB	0.450	0.50	0	1					
Wife’s SWB = husband’s SWB	0.088	0.28	0	1					
Wife’s SWB < husband’s SWB	0.463	0.50	0	1					
Preferences									
Trust	0.400	0.13	0.1	0.9	0.388	0.12	0.1	0.6	0.013
Reciprocity	0.384	0.22	0.1	1.0	0.360	0.18	0.1	1.0	0.023*
Altruism	0.380	0.11	0.1	0.7	0.386	0.12	0.1	0.6	−0.006
Risk lovingness	0.423	0.12	0.2	1.0	0.390	0.12	0.1	0.6	0.033
Individual characteristics									
Age	29.4	6.7	18	47	38.1	11.6	18	72	−8.7***
Education: none	0.450	0.50	0	1	0.375	0.49	0	1	0.075
Education: primary	0.250	0.44	0	1	0.213	0.41	0	1	0.038
Education: middle	0.250	0.44	0	1	0.313	0.47	0	1	−0.063
Education: higher	0.050	0.22	0	1	0.100	0.30	0	1	−0.050
Religion: Catholic	0.338	0.48	0	1	0.288	0.46	0	1	0.050
Religion: Muslim	0.225	0.42	0	1	0.213	0.41	0	1	0.013
Religion: Charisma	0.088	0.28	0	1	0.075	0.27	0	1	0.013
Religion: traditional African	0.075	0.27	0	1	0.188	0.39	0	1	−0.113***
Religion: others	0.275	0.45	0	1	0.238	0.43	0	1	0.038
Couple’s or household’s characteristics									
Married (years)	8.8	6.3	1	25					
Number of children	3.9	2.3	1	10					
Asset: lowest	0.263	0.44	0	1					
Asset: lower middle	0.238	0.43	0	1					
Asset: upper middle	0.250	0.44	0	1					
Asset: highest	0.250	0.44	0	1					

*p < 0.1, *** *p* < 0.01 obtained through a paired *t* test (Wilcoxon signed-rank test for subjective well-being)

### Happiness and preferences

According to Table 1, the mean score for wives’ subjective well-being (5.6) was lower than that for husbands (5.8), although statistical significance was not confirmed by Wilcoxon signed-rank tests. Regarding happiness, 45.0% of the wives felt happier than their husbands, and 8.8% of the wives felt the same level of happiness as their husband.

With respect to preferences, the mean trust score was 0.40 among wives and 0.388 among husbands. At the 10% significance level, the mean reciprocity score was higher among wives (0.384) than among husbands (0.360). The mean altruism score was 0.380 among wives and 0.386 among husbands. Finally, the mean for risk lovingness was 0.423 among wives and 0.390 among husbands.

### Regression analysis

Table 2 reports the estimated results from happiness equations for wives and husbands. The absolute gap within couples in regard to risk lovingness was associated with wives’ higher levels of happiness (OR = 4.83), while the absolute gaps in regard to trust and altruism were associated with husbands’ lower levels of happiness (OR = −3.58 for trust, OR = −3.33 for altruism). Table 2 also presents the effects of wives’ and husbands’ basic characteristics on levels of happiness. The results show that, as illustrated in column 1, wives’ ages were negatively associated with their subjective well-being (OR = −0.09) and that Charismatic belief was associated with a higher level of happiness (OR = 1.89). Additionally, column 5 shows that husbands with higher levels of education had higher levels of happiness (OR = 1.70). The number of children was negatively correlated with husbands’ happiness levels, at less than the 1% significance level, and it was not statistically significant in regard to wives’ happiness.

**Table 2:** Results of regression analyses regarding individual happiness

	Wife’s happiness				Husband’s happiness			
	1	2	3	4	5	6	7	8
Trust: absolute gap	0.71 (0.78)				−3.58 (0.04)**			
Trust: wife > husband	−0.24 (0.66)				−0.58 (0.25)			
Reciprocity: absolute gap		2.27 (0.27)				0.18 (0.89)		
Reciprocity: wife > husband		0.29 (0.57)				0.75 (0.31)		
Altruism: absolute gap			3.08 (0.40)				−3.33 (0.02)**	
Altruism: wife > husband			0.18 (0.84)				−0.09 (0.85)	
Risk lovingness: absolute gap				4.83 (0.08)*				−2.12 (0.40)
Risk lovingness: wife > husband				0.44 (0.58)				0.70 (0.15)
Age	−0.09 (0.04)**	−0.08 (0.10)*	−0.09 (0.02)**	−0.07 (0.23)	0.02 (0.33)	0.04 (0.12)	0.04 (0.15)	0.04 (0.08)*
Education: none^a^								
Education: primary	−0.53 (0.29)	−0.44 (0.36)	−0.31 (0.65)	−0.63 (0.29)	1.56 (0.03)**	1.45 (0.02)**	1.52 (0.02)**	1.60 (0.01)**
Education: secondary	−0.35 (0.71)	−0.21 (0.81)	−0.25 (0.78)	−0.30 (0.75)	1.06 (0.11)	0.79 (0.16)	0.90 (0.17)	0.75 (0.28)
Education: higher	−0.44 (0.85)	−0.62 (0.84)	−0.17 (0.95)	−0.48 (0.86)	1.70 (0.09)*	1.67 (0.11)	1.67 (0.08)*	1.39 (0.19)
Religion: Catholic^a^								
Religion: Muslim	−0.08 (0.93)	0.04 (0.96)	−0.16 (0.88)	0.08 (0.94)	0.23 (0.77)	−0.21 (0.78)	0.30 (0.65)	−0.18 (0.79)
Religion: Charisma	1.89 (0.03)**	1.52 (0.11)	1.47 (0.17)	1.52 (0.06)*	0.95 (0.36)	0.74 (0.52)	1.04 (0.31)	0.74 (0.58)
Religion: traditional African	−0.79 (0.32)	−0.82 (0.32)	−1.11 (0.13)	−1.02 (0.21)	0.53 (0.65)	0.18 (0.88)	0.65 (0.50)	0.27 (0.78)
Religion: others	0.80 (0.31)	0.87 (0.24)	0.75 (0.29)	0.73 (0.38)	0.15 (0.86)	−0.23 (0.81)	0.12 (0.89)	0.06 (0.95)
Married (years)	0.02 (0.66)	0.03 (0.44)	0.02 (0.71)	0.02 (0.71)	0.08 (0.06)*	0.07 (0.07)*	0.08 (0.08)*	0.07 (0.12)
Number of children	0.02 (0.85)	−0.02 (0.81)	0.05 (0.68)	0.03 (0.67)	−0.32 (0.00)***	−0.39 (0.00)***	−0.37 (0.00)***	−0.37 (0.00)***
Asset: lowest^a^								
Asset: lower middle	−0.33 (0.54)	−0.59 (0.25)	−0.46 (0.38)	−0.07 (0.92)	−0.22 (0.76)	0.14 (0.84)	−0.09 (0.91)	0.01 (0.99)
Asset: upper middle	1.44 (0.16)	1.11 (0.12)	1.24 (0.17)	1.48 (0.05)**	0.14 (0.79)	0.36 (0.52)	0.15 (0.78)	0.06 (0.92)
Asset: highest	0.00 (1.00)	0.10 (0.81)	0.03 (0.94)	0.23 (0.57)	0.91 (0.20)	1.14 (0.25)	0.80 (0.32)	0.94 (0.33)

Estimated coefficients are adjusted proportional odds ratios (OR); *p* value in parenthesis

*p < 0.1, **p < 0.05, ***p < 0.01

^a^Reference group

Table 3 reports the estimated results from the multivariable logistic and ordered-logistic regressions. Panel A presents the effects of the absolute gap in preferences on the likelihood that wives have higher levels of happiness than their husbands. The results show that all of the absolute gaps in preferences are positively associated with this likelihood, with a statistical significance for trust (OR = 6.22), reciprocity (OR = 2.80), and risk lovingness (OR = 3.81). Panel B reports the effects the within-couple gap in preferences has on the within-couple gap in subjective well-being. Similarly, all the within-couple gaps in preferences were positively related to the happiness gap, with a statistical significance for trust (OR = 4.77) and risk lovingness (OR = 3.72).

**Table 3:** Results of multivariate regression analyses

	A. Logistic regression				B. Ordered-logistic regression			
	If wife’s SWB > husband’s SWB				Wife’s SWB-husband’s SWB			
Trust: absolute gap	6.22 (0.01)**				4.77 (0.09)*			
Trust: wife > husband	−0.22 (0.81)				−0.25 (0.59)			
Reciprocity: absolute gap		2.80 (0.01)***				1.12 (0.60)		
Reciprocity: wife > husband		0.89 (0.14)				0.48 (0.25)		
Altruism: absolute gap			6.38 (0.26)				6.38 (0.11)	
Altruism: wife > husband			−0.11 (0.92)				0.03 (0.97)	
Risk lovingness: absolute gap				3.81 (0.07)*				3.72 (0.07)*
Risk lovingness: wife > husband				0.59 (0.58)				0.64 (0.29)

Covariates: age, education level, religion of both the wife and the husband, years of marriage, number of children, asset quartile; estimated coefficients constitute adjusted odds ratios for “A. logistic regression” and adjusted proportional odds ratios for “B. ordered-logistic regression”; *p* value in parenthesis

*p < 0.1; **p < 0.05; ***p < 0.01

## Discussion

This is the first study to examine the association between preference gaps and happiness status for married couples in the afterbirth period using matched couples and experimental data. This study showed that the gap in preferences between a couple is closely associated with the happiness of wives and husbands in the afterbirth period. Furthermore, the preference gap between a couple had a positive effect on women’s relative happiness with their husbands.

Wives’ happiness levels were positively associated with the absolute value of the gap in preferences, particularly in regard to risk lovingness. This suggests that wives felt happier if they were greater risk-takers than their husbands or if their husbands were more risk-averse than them. One possible reason for this result is that wives do not want their husband to engage in risky behavior or to make risky decisions in the course of supporting their family. However, even in cases where their risk-taking levels are lower than that of their husbands, wives can still accept and maintain their happiness levels. On the contrary, husbands’ happiness was negatively related to the preference gap, especially in relation to trust and altruism. This means that husbands were more likely to be happier if the couples shared similar preferences in regard to trust and altruism. Our study also confirmed that the preference gap between a couple had a positive effect on women’s relative happiness with their husbands. These results indicate that women feel greater happiness than their spouses if they do not share similar preferences.

Recently, more attention has been paid to the gender gap in regard to happiness [24], especially among married couples [11, 12]. Importantly, it was shown that if a wife is unhappier than her husband, the couple are more likely to get a divorce [11]. Nonetheless, very few studies have examined the determinants of the happiness gap between a couple, with the exception of the study of Posel and Casale, which was conducted in South Africa [25]. Therefore, our analysis will provide new findings on the within-couple happiness gap.

Importantly, the above results were obtained after controlling in the multivariate regressions for the number of years married, suggesting the effects of the within-couple preference gap on happiness were valid regardless of the length of marriage. This indicates the possibility that, at the time of marriage, couples choose partners who have similar preferences, i.e., “assortative mating,” rather than that the couples have been affected by sharing the same living environment after marriage. Previous studies have investigated assortative mating in regard to educational attainments [26–29], physical traits [30–33], and health status [34, 35].

Nevertheless, only a limited number of studies have examined whether or not assortative mating exists in regard to preferences. For instance, for German couples, a positive correlation in regard to risk preference has been found. However, this correlation increased with years of marriage, which indicates the effect of *socialization* (the tendency that the preferences of the wife and husband become similar to years of marriage) rather than assortative mating [36]. In another study, positive spousal correlations in cooperation and altruism were found using a sample of married couples from rural communities in Senegal. They found this correlation was due to assortative mating at the time of marriage rather than influence after marriage [37]. In line with these studies, our study showed that the happiness of wives and husbands corresponds to their gap in preferences, regardless of the length of marriage. This result will also give a new insight into the debate about assortative mating in regard to preferences.

Finally, regarding the effects of couple’s characteristics on happiness, it was shown that husbands’ happiness levels lower as the number of children increases. This is consistent with the results of previous studies, which have determined that increases in the presence and number of children lessen parents’ happiness [38, 39]. As rearing a child is both rewarding and burdensome for parents, the family context has a strong effect on whether parenthood has a positive or negative influence on the parents’ happiness [39]. Parents can be happier when the marginal benefits of parenting outweigh the marginal cost. To examine this point in more detail, we analyzed our data to determine how parents’ happiness levels differed depending on the number of children in each family. Consequently, we found that husbands’ happiness levels decreased if the number of children exceeded four; in contrast, wives’ happiness levels did not decline as the number of children increased. This contrast can be partly explained by the different parental responsibilities within the family. Fathers usually take financial responsibility for the family and, therefore, they tend to feel more stress than mothers if the financial burden rises following an increase in the number of children in the family [39].

We also confirmed the contrasting influence a traditional African religion has on wives and husbands. Regression results showed that believing in a traditional African religion was associated with lower happiness levels than Catholic believers for wives, but not for husbands. Since religion is closely related with one’s subjective well-being [21], further investigation is required to clarify how religion affects the happiness of married couples in Ghana.

Our research was not without limitations, an example of which is that the direction of causality from the preference gap to happiness was not fully confirmed because we only used cross-sectional data. Second, we could not identify the concrete reasons behind the significant effects of gender-preference gaps on happiness. A detailed investigation into the process from couple’s preference gaps to their happiness would be a valuable resource for future research.

## Conclusions

Our results showed that a wider gap in preferences was associated with wives’ happiness relative to that of their husbands. To prevent postnatal depression following childbirth, the effect of the gap on preferences must be more focused.

## Abbreviations

BMI: Body mass index
EMBRACE: Ensure Mothers and Babies Regular Access to Care
KHDSS: Kintampo Health Demographic Surveillance System
